# Understanding the antimicrobial activity of selected disinfectants against methicillin-resistant *Staphylococcus aureus* (MRSA)

**DOI:** 10.1371/journal.pone.0186375

**Published:** 2017-10-16

**Authors:** Ebrahim Aboualizadeh, Violet V. Bumah, Daniela S. Masson-Meyers, Janis T. Eells, Carol J. Hirschmugl, Chukuka S. Enwemeka

**Affiliations:** 1 Physics Department, University of Wisconsin-Milwaukee, Milwaukee, Wisconsin, United States of America; 2 Department of Chemistry and Biochemistry, College of Sciences, San Diego State University, San Diego, California, United States of America; 3 Department of Biomedical Sciences, College of Health Sciences, University of Wisconsin- Milwaukee, Milwaukee, Wisconsin, United States of America; 4 Office of provost, San Diego State University, San Diego, California, United States of America; Tallinn University of Technology, ESTONIA

## Abstract

Disinfectants and biocidal products have been widely used to combat Methicillin-resistant *Staphylococcus aureus* (MRSA) infections in homes and healthcare environments. Although disruption of cytoplasmic membrane integrity has been documented as the main bactericidal effect of biocides, little is known about the biochemical alterations induced by these chemical agents. In this study, we used Fourier transform infrared (FT-IR) spectroscopy and chemometric tools as an alternative non-destructive technique to determine the bactericidal effects of commonly used disinfectants against MRSA USA-300. FTIR spectroscopy permits a detailed characterization of bacterial reactivity, allowing an understanding of the fundamental mechanism of action involved in the interaction between bacteria and disinfectants. The disinfectants studied were ethanol 70% (N = 5), isopropanol (N = 5), sodium hypochlorite (N = 5), triclosan (N = 5) and triclocarban (N = 5). Results showed less than 5% colony forming units growth of MRSA treated with triclocarban and no growth in the other groups. Nearly 70,000 mid-infrared spectra from the five treatments and the two control (untreated; N = 4) groups of MRSA (bacteria grown in TSB and incubated at 37°C (Control I) / at ambient temperature (Control II), for 24h) were pre-processed and analyzed using principal component analysis followed by linear discriminant analysis (PCA-LDA). Clustering of strains of MRSA belonging to five treatments and the discrimination between each treatment and two control groups in MRSA (untreated) were investigated. PCA-LDA discriminatory frequencies suggested that ethanol-treated spectra are the most similar to isopropanol-treated spectra biochemically. Also reported here are the biochemical alterations in the structure of proteins, lipid membranes, and phosphate groups of MRSA produced by sodium hypochlorite, triclosan, and triclocarban treatments. These findings provide mechanistic information involved in the interaction between MRSA strains and hygiene products; thereby demonstrating the potential of spectroscopic analysis as an objective, robust, and label-free tool for evaluating the macromolecular changes involved in disinfectant-treated MRSA.

## Introduction

Drug resistant microorganisms pose serious challenges to the development of effective treatment regimens. Methicillin-resistant *Staphylococcus aureus* (MRSA), a Gram-positive bacterium, is one of such microbes of global health concern. Various antiseptics and disinfectants; including alcohols, aldehydes, biguanides, peroxygens, phenols and cresols, have been used against nosocomial infections in shared facilities [1–4]. Moreover, their effects on bacterial metabolism have been studied by various methods, including examination of their uptake [5], lysis and leakage of intracellular constituents [6], perturbation of cell homeostasis and membrane [7,8], inhibition of enzymes, electron transport and oxidative phosphorylation [9,10], and interaction with macromolecules [11, 12], amongst others. However, much remains unknown about the antimicrobial mechanisms of these biocides.

The antimicrobial effect of hand hygiene agents against MRSA, such as carbohydrate fatty acid derivatives and various antiseptics has been studied by various groups [13–16]. Ethyl alcohol (ethanol) and isopropyl alcohol (isopropanol) are known to be antiseptically most effective against spores [17,18]. Although cell membrane damage is considered as the major antimicrobial activity of alcohols, biochemical changes in the specific macromolecules needs to be revealed. Sodium hypochlorite or household bleach, a chlorine-releasing agent commonly used for disinfection, has been shown to alter proteins, DNA and cell membrane [19–22]. However, the underlying mechanism of its action is not clear. The same is true for Triclosan (2,4,4/-trichloro-2/-hydroxydiphenyl ether), a derivative of phenolic groups, and Triclocarban (TCC; 3,4,49-triclorocarbanilide); both of which are widely used household hygiene products with broad-spectrum efficacy against gram-positive bacteria, including MRSA strains [23,24].

FTIR spectroscopy is an analytical technique that relies on the detection of characteristic molecular vibrations in a biological sample attributed to proteins, lipids, nucleic acids, and others without applying any dyes or stains. Over the past years, FTIR spectroscopy together with multivariate analysis has been extensively used for assessing thousands of spectra for biomarker extraction [25,26], delineating the cells hierarchy [27–29], and the structure of tissues in the microscopic realm [30–33]. The ability of FTIR spectroscopy and statistical analyses, particularly principal component analysis, hierarchical clustering analysis, and artificial neural network in identifying bacteria and taxonomical discrimination between species has been thoroughly investigated [34–38].

Recently, we successfully used a combination of FTIR spectroscopy and chemometric tools to examine the antimicrobial mechanisms of blue light (470 nm) on MRSA [39]. As a follow up in this study, we demonstrate for the first time that the same technique can be used to uncover the antimicrobial effects of ethanol, isopropanol, sodium hypochlorite, triclosan and triclocarban on MRSA, and report on the biomolecular changes involved. We present the biomarkers (discriminatory frequencies) derived from clustering between the control (untreated) MRSA and the disinfectant-treated MRSA to reveal the mode of action of the disinfectants against bacteria.

## Materials and methods

**Fig 1:**
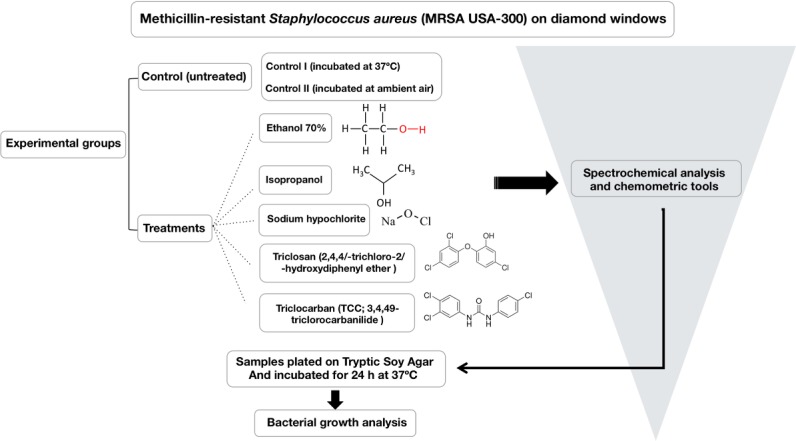
Schematics of the experimental design.

Five different biocide treated groups and two control (non-treated bacteria) groups were evaluated using FTIR spectroscopy. Bacteria were treated with ethanol (N = 5), isopropanol (N = 5), sodium hypochlorite (N = 5), triclosan (N = 5) and triclocarban (N = 5); while the group control I, comprised of untreated MRSA USA-300 incubated at 37°C for 24 hours (N = 4); and control II was comprised of untreated MRSA USA-300 incubated in ambient air for 24 hours (N = 4); The rationale for control II was to determine if there would differences between bacteria left to die through a natural process (devoid of adequate growth environment) as opposed to treatment with chemicals. Schematics of the experimental design are shown in Fig 1.

### Bacterial culture

MRSA USA-300 strain (ATCC® BAA-1680) was purchased from American Type Culture Collection (ATCC) (Manassas, VA, USA) and identified by standard procedures; including PCR. USA-300 strain is the most predominant Community associated MRSA strain in the United States, and this strain has been fully sequenced [40]. For the experiments, a colony of MRSA USA-300 grown in trypticase soy broth (TSB) at 37°C for 24 hours was centrifuged at 13000 rpm for 5 minutes, supernatant discarded and pellet re-suspended in different concentrations of biocides for 30 minutes. Aliquots of 2μl of 2×10^9^ CFU/mL were placed on diamond optical windows (size 5.5 mm diameter-600 μm thick) for FTIR analysis and then incubated at 37°C for 24 hours for colony assay. This article does not contain any studies with human participants or animals performed by any of the authors.

### Treatment of MRSA with biocides

#### Ethyl alcohol, isopropyl alcohol and sodium hypochlorite treatment

A volume of 10 mL of MRSA grown overnight was centrifuged at 13000g for 5 minutes. The supernatant was removed leaving approximately 10 μl of TSB in the tube for bacteria re-suspension. 200 μl of 70% alcohol, 70% isopropyl alcohol and 5% bleach were added to separate tubes containing 2x10^9^ CFU/mL MRSA and 2 μl pipetted on different diamond windows for FTIR measurements respectively. After imaging, the contents of the windows were swabbed onto 3 separate TSA plates and incubated at 37°C for 24 hours.

#### Triclosan and triclocarban treatment

A volume of 10 mL of MRSA grown overnight was centrifuged at 13000g for 5 minutes. The supernatant was removed leaving approximately 10 μl of TSB in the tube for bacteria re-suspension. 200 μl of 1% triclosan (AcuStandard, Inc. New Haven, USA; Stock solution (100 mg/mL) is dissolved in ethanol) was added to the tube containing 2×10^9^ CFU/mL MRSA and 2 μl pipetted on to a diamond windows for FTIR measurements. Likewise, 200 μl of a Minimum Inhibitory Concentration of 8 mg/mL of triclocarban (Crescent Chemical Co., Inc. New York, USA) was added to a tube containing 2×10^9^ CFU/mL MRSA and 2 μl pipetted on to a diamond window for FTIR measurements. After imaging, the contents of the windows were swabbed onto 2 separate TSA plates and incubated at 37°C for 24 hours.

### FT-IR spectroscopy and data pre-processing

IR spectra of disinfectant-treated and the control MRSA mounted on diamond optical windows were collected in transmission mode using a Bruker Vertex 70 IR spectrometer and a Bruker Hyperion 3000 IR Microscope. The setup was attached to a liquid nitrogen-cooled focal point array (FPA) detector and a computerized x-y stage. Standard 36× Cassegrain microscope objective and a 15× Cassegrain condenser were used, which provided 1.1 μm pixel resolution and a 70 × 70 μm^2^ FOV. The spectra from FPA-FTIR measurements in the wavenumber range 900–3800 cm^-1^ were collected with 1024 co-added scans and 4 cm^-1^ spectral resolution. Background measurements were from a clean (no sample) area on the diamond window and the ratio of sample measurement to this background was evaluated as an absorption spectrum. The contribution of each disinfectant to the absorption spectrum of MRSA was evaluated by measuring pure references and it was negligible (less than 5% absorbance). The FPA size was set to 64×64 for the measurements; therefore, 4096 spectra were generated from each measurement. 4×4 pixel binning was applied to the spectra to obtain higher signal to noise ratio and more accurate classification. Data pre-processing was carried out after data acquisition using Igor Pro 6.4 and Matlab_R2016a as follows. CO_2_ peak at 2350 cm^-1^ was flattened between 2500 and 2200 cm^-1^ and then baseline was corrected by finding a least squares linear line between all spectral points in 2692–1920 cm^-1^ spectral region and subtracted from every spectrum. Then, signal to noise ratio (S/N) in every spectrum was systematically assessed by defining the noise content as the standard deviation in the 2000–1900 cm^-1^ spectral region, and the signal as the maximum of the curve between 1700 and 1600 cm^-1^ (Amide I band). Then, the spectra were cut into the biochemical-IR spectral region 1800–900 cm^-1^ (n = 467 data points per spectrum) prior to multivariate analysis.

### Multivariate data analysis

PCA is an unsupervised method that provides a set of principal components representing modes of maximum variance within the data set, as well as a set of scores describing where the original data points lie in the space defined by the principal components. Plotting these scores enables visualization of the underlying similarities and differences among data points in a low-dimensional space. PCA loadings plot highlights the major variables (wavenumbers) that contribute the most to the total variance in the data, described by that principal component. Linear discriminant analysis (LDA) is a supervised method that is applied posterior to PCA to maximize the inter-group variations than intra-group variations. A *priori* information from principal components (PC) was used as an input to the LDA algorithm to attain the maximum classification between clusters (LD factors). The optimum number of PCs was retained based on iterative process of validating prior to LDA algorithm. For each cluster, a cluster vector plot was derived from LDA, which highlights the biomarkers and the pathway describing the response of the bacteria to each treatment [41, 42]. From each group of control and treated bacteria, we derived spectra from each pixel of the FPA-FTIR measurements (nearly 10000 spectra) and pre-processed for importing into PCA algorithm. Each spectrum represented an area of 1.1×1.1 μm^2^ in the window (i.e. every pixel within an FPA-FTIR measurement contains an FTIR spectrum). The computational analysis was performed using “python 2.7.11” and “R” (version 3.1.2) software.

### Colony forming units (CFU)

**Fig 2:**
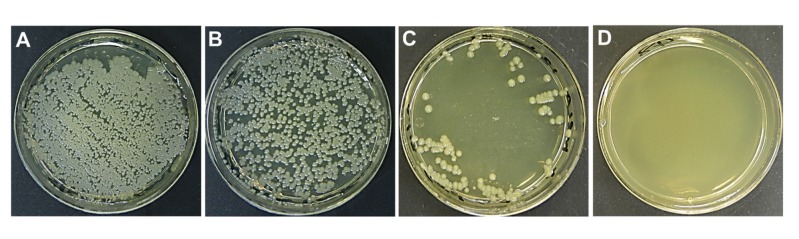
Representative culture plates of MRSA. (A) Control I. (B) Control II. (C) Triclocarban-treated sample. (D) Representative plate showing no bacterial growth from samples treated with either Ethanol, Isopropanol, Sodium hypochlorite or Triclosan.

Colony-forming unit (CFU) is a measure of viable bacteria and the results are presented as CFU/mL for each plate. Bacterial growth as determined by counting the CFU on each plate, assesses the effect of detergent/disinfectant on bacteria growth before and after FTIR measurements. A swab was dipped in sterile saline and rolled over the diamond windows of the group tested at the time. The swab was then used to streak tryptic soy agar (TSA) plates and incubated at 37°C for 24 hours. The pictures of plates were taken and colonies enumerated. This procedure was repeated for all groups of disinfectants- and antiseptics-treated MRSA. Fig 2A and 2B show the CFU results for both of the control groups and the results confirm that the infrared signals observed for these two groups are indicative of a functioning MRSA. The bacterial colonies were clearly visible for the control I and the control II groups and the difference observed between Fig 2A and 2B comes from the density of bacteria after swabbing the windows. Fig 2C shows the triclocarban-treated MRSA with a growth of less than 5% after incubation at 37°C for 24 hours. The ethanol-, isopropanol-, sodium hypochlorite- and triclosan-treated samples did not show any bacterial growth (Fig 2D).

## Results and discussion

The central premise of this study was designed to identify the biochemical alterations of disinfectants-treated MRSA. Thus, PCA-LDA was used to derive scores plot and vector plot from making comparisons between the combination of treatments and control groups. PCA-LDA vector plot was used to determine the biomarkers that indicate the biochemical alterations in macromolecules toward death that each treatment protocol takes. Classification between spectra using PCA-LDA was performed for three classes of spectra: 1) comparison between all treatments (excluding the control groups), 2) comparison between the control (untreated) groups and the alcohol-treated (ethanol- and isopropanol-treated) MRSA, and 3) comparison between the control (untreated) groups and the disinfectant-treated (sodium hypochlorite-, the triclosan-, and the triclocarban-treated) MRSA. The main reason behind performing PCA-LDA on individual treatments and control groups is to provide more accurate biochemistry attributed to each treatment. The focus of this study is not to have a comparative minimum inhibitory concentration (unit) of biocides or a time course of exposure, but to have a dose that maximally inactivates the bacteria, and to monitor using FTIR possible signatures of macromolecules, which might indicate the mechanism toward death that each treatment protocol takes.

### Classification between biocides applied to MRSA

**Fig 3:**
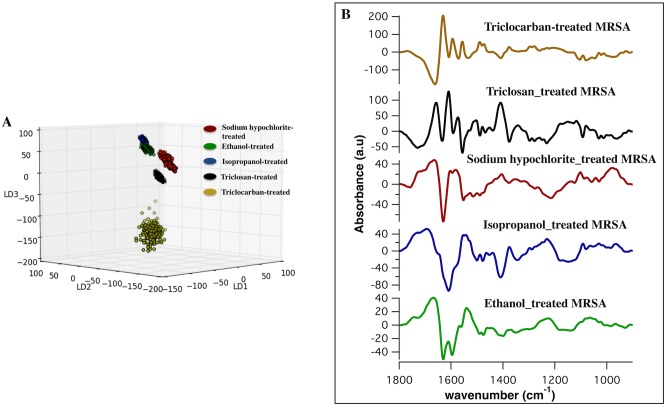
PCA-LDA scores and loadings plot from comparison of the ethanol-treated, isopropanol-treated, sodium-hypochlorite-treated, triclosan-treated and triclocarban-treated spectra. A) Three-dimensional scores plot of IR spectra from all treated-MRSA groups, which clearly show the separation of spectra. Each point in the scatter plot represents a pixel IR spectrum (an area of 1.1×1.1μm^2^). B) Corresponding cluster vector plot for each treatment showing the discriminatory features in the spectral region between 1800 and 900 cm^-1^, responsible for the separation of spectra; i.e. triclocarban-treated (yellow); triclosan-treated (black); sodium hypochlorite-treated (red); isopropanol-treated (blue) and ethanol-treated (green), respectively. Nearly 10,000 IR spectra were derived from each group for the comparison.

Spectra from five groups of samples that were treated by multiple biocides were classified and the biochemical markers attributed to each treatment were achieved. The PCA-LDA model is built based on revealing similarities and differences between the classes and the classification of the spectra are clearly visible in the three-dimensional scores plot (Fig 3A). As shown (Fig 3A), the triclocarban-treated group was extensively more diverse as compared to the tight cluster of scores associated with the other treatments. Along LD1, there was a separation between the triclocarban-treated spectra and the spectra from the rest of the groups and along LD2; the isopropanol- and the ethanol-treated spectra segregated from the sodium hypochlorite-treated and the triclosan-treated spectra. Along LD3, there was a separation between spectra from the hypochlorite-treated group and the ethanol, the isopropanol, and the triclosan-treated group. Scores plot (Fig 3A) reveals that the ethanol and the isopropanol-treated spectra are the closest adjacent clusters, which suggest the intimacy of the biomolecular signatures of these biocides with regards to the rest of the treatments. The list of the IR bands from PCA-LDA (discriminatory frequencies) and associated biomolecular assignments attributed to each treatment is summarized in Table 1.

**Table 1:** Discriminatory frequencies (cm^-1^) of the ethanol-treated, isopropanol-treated, sodium hypochlorite-treated, triclosan-treated and triclocarban-treated MRSA derived from PCA-LDA.

Ethanol-treated (cm^-1^)	Isopropanol-treated (cm^-1^)	Sodium hypochlorite-treated (cm^-1^)	Triclosan-treated (cm^-1^)	Triclocarban-treated (cm^-1^)	Molecular Assignment
	1695				Proteins: Antiparallel *β*-sheet (Amide I)
1666		1664		1662	Proteins: turns 3_10_ helical structure (Amide I)
			1656		Proteins: α-helical structure (Amide I)
1629		1629	1633	1629	Proteins: *β*-sheet structure (Amide I)
	1608		1608	1608	Proteins: aggregated strands (Amide I)
1594		1594	1591	1594	Proteins: mainly N-H bending (Amide II)
			1573	1571	Proteins: mainly NH bending (Amide II)
	1552	1552	1556	1556	Proteins: mainly N-H bending (Amide II)
1540					Proteins: *α*-helical structure (Amide II)
			1488	1488	Lipids: C-H deformation
			1467	1469	Lipids: CH_2_ bending/CH_3_ deformation
	1409	1407	1409	1407	Lipids: C-H deformation
	1375	1377	1375	1377	Lipids: CH_3_ deformation
	1230	1216			Phosphates: ν_asym_ PO^2-^
		1101			Phosphates: ν_sym_ PO^2-^
	1091		1093	1091	Phosphates: ν_sym_ PO^2-^

As shown (Fig 3B), the cluster vector plot highlights that the most important differences between the treatments were based in amide I and amide II of β-sheet, α-helical, and aggregated strands protein structures. Further differences were present in the C-H deformations of lipids and stretches of phosphates. The changes in the protein profile in triclosan-treated and triclocarban-treated MRSA may be related to the specific interaction of the phenolic compound with an enoyl-acyl reductase carrier protein [43–46]. The similarities of several proteins, lipids, and nucleic acids bands in the loading plot attributed to triclosan and triclocarban-treated groups (Table 1), suggest the similar biochemical alterations in MRSA induced by these biocides. This important finding is in agreement with several studies that hypothesized triclocarban’s molecular mechanism resembles that of triclosan [47]. When sodium hypochlorite-treated spectra were compared with the other treatments, the biochemical changes in the protein structure were somewhat similar to that of triclosan and triclocarban; however, some protein bands experienced slight shifts.

### Control (untreated) MRSA *vs*. alcohol-treated MRSA

**Fig 4:**
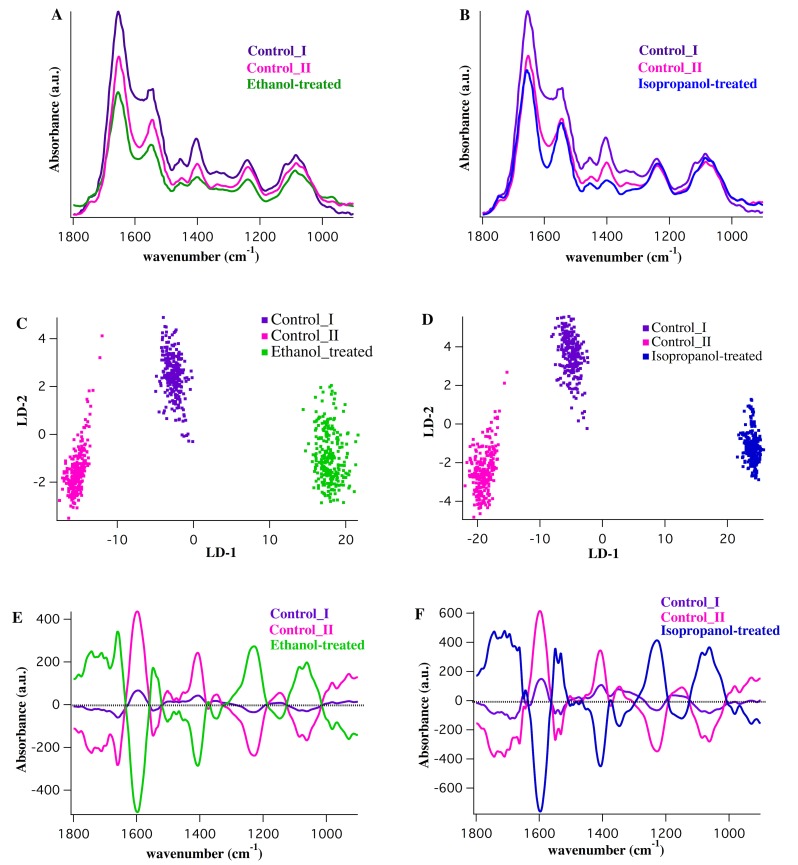
Comparison of the ethanol-treated and isopropanol-treated MRSA *vs*. control groups. A,B) Average IR spectra from control I (purple), control II (pink), ethanol-treated (green) and isopropanol-treated (blue) MRSA. The spectra were averaged over nearly 40,000 individual IR spectra (64×64 pixels; 4096 spectra per measurement, 10 measurement per sample). C,D) Two-dimensional scores plot from PCA-LDA analysis (LD-2 *vs*. LD-1) derived from the comparison between control I, control II and ethanol-treated spectra (C) and between the controls and isopropanol-treated spectra (D). E,F) Corresponding cluster vector plot for the comparison made in C,D and the vector plot per cluster showing the significant discriminatory features, is shown.

Fig 4A and 4B show the mid-infrared absorption spectra in the spectral range between 1800 and 900 cm^-1^ derived from the control I, the control II, the ethanol-treated and the isopropanol-treated MRSA, respectively. Fig 4C and 4D show the two-dimensional scores plots (LD2 *vs*. LD1) derived from the clustering of the ethanol-treated and the isopropanol-treated spectra with respect to the control spectra. Each treatment was compared individually with the two control groups. There was a clear separation between the spectra along LD1, which distinctively classified the control I and the control II spectra from the ethanol-treated and the isopropanol-treated spectra. As shown (Fig 4E and 4F), vector plot for the isopropanol-treated group reveals similar pattern as the ethanol-treated group, although there are several differences in protein bands and shifts in a few wavenumbers. Spectral biomarkers from the comparison between the ethanol-treated and the isopropanol-treated MRSA with the control groups are listed in Table 2.

**Table 2:** Discriminatory frequencies (cm^-1^) of the comparison between ethanol-treated and isopropanol-treated, with control groups of MRSA derived from PCA-LDA.

Ethanol-treated (cm^-1^)	Isopropanol-treated (cm^-1^)	Molecular Assignment
1683	1683	Proteins: Amide I/turns or antiparallel β-sheet structure
1660	1660	Proteins: Amide I/turns 3_10_ helical structure
1597	1597	Proteins: Amide II
1548	1550	Proteins: Amide II/mainly N-H bending; α-helical structure
	1531	Proteins: Amide II/mainly N-H bending; β-sheet structure
1407	1407	Lipids: C-H deformation
	1346	Lipids: C-H deformation
1228	1228	Phosphates: ν_asym_ PO^2-^
1085	1085	Phosphates: ν_sym_ PO^2-^
1062	1062	Carbohydrates: C-O stretching; DNA/RNA

The most conspicuous differences were present in the amide I band components resulting from antiparallel β-sheets, and 3_10_ helical turns of proteins as well as amide II of α-helical and β-sheet secondary structure of proteins. C-H deformations in lipids, C-O stretches dominated by ring vibrations of carbohydrates, DNA/RNA, and P = O stretches in phosphates were additional signatures of damage caused by ethanol and isopropanol. Little is known about the specific mode of action of alcohols in microorganisms; however, protein denaturation and disruption of membranes [48–50] are the most well known effects of ethanol and isopropanol against pathogens. Our findings suggest that the targets of interaction between MRSA cells and alcohols are mainly in the structure of proteins and the phosphates. These findings provide a more detailed understanding of the biochemical changes in the proteins embedded in cytoplasmic membrane and the phosphates in the gram-positive bacterial cell wall. As demonstrated, the ethanol- and isopropanol-induced biochemical changes in MRSA cells are reported; however, molecular methodologies are needed to determine more biologically intrinsic details and an intricate event-driven process chains.

### Control (untreated) MRSA *vs*. disinfectant-treated MRSA

**Fig 5:**
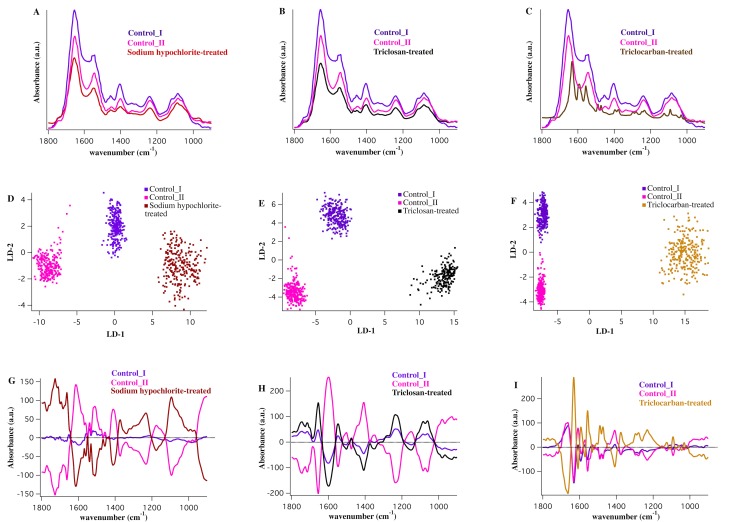
Comparison of the sodium hypochlorite-treated, triclosan-treated and triclocarban-treated spectra *vs*. control I and control II groups. A,B,C) Average IR spectra from control I (purple), control II (pink), sodium hypochlorite-treated (red), triclosan-treated (black) and triclocarban-treated (yellow) MRSA. The spectra were averaged over nearly 40,000 individual IR spectra in each case. D,E,F) Two-dimensional scores plot from PCA-LDA analysis (LD-2 *vs*. LD-1) derived from the comparison of control I, control II and sodium hypochlorite-treated (D), triclosan-treated (E) and triclocarban-treated (F). G,H,I) Corresponding cluster vector plot for the comparison made in D, E and F and the vector plot per cluster highlighting the significant discriminatory features, is demonstrated.

The average mid-infrared absorption spectra within the spectral range 1800-900cm^-1^ derived from the sodium hypochlorite-treated, the triclosan-treated, the triclocarban-treated, and the control groups are shown (Fig 5A, 5B and 5C). The chemistry of the sodium hypochlorite, the triclocarban and the triclosan is comprehended by FTIR measurements on pure disinfectants (data not shown). Two-dimensional PCA-LDA scores plot and the corresponding cluster vector plots from the comparison of each individual disinfectant treatment with the control groups are shown in Fig 5D–5I. Discriminatory frequencies from the comparison between the triclosan-, the sodium hypochlorite-, and the triclocarban-treated MRSA and the control groups are listed in Table 3.

**Table 3:** Discriminatory frequencies (cm^-1^) of the comparison between sodium hypochlorite-treated, triclosan-treated and triclocarban-treated, and control groups of MRSA derived from PCA-LDA.

Sodium hypochlorite-treated (cm^-1^)	Triclosan-treated (cm^-1^)	Triclocarban-treated (cm^-1^)	Molecular Assignment
1735	1743		Lipids: C = O ester in phospholipids
	1685		Proteins: Amide I/turns or antiparallel β-sheet structure
1662		1662	Proteins: Amide I/turns 3_10_ helical structure
	1654		Proteins: Amide I/α-helical structure
		1631	Proteins: Amide I/ mainly C = O stretching, β-sheet structure
1614		1608	Proteins: Amide I/ aggregated strands
	1598	1593	Proteins: Amide II
		1556	Proteins: Amide II/ mainly N-H bending
1539			Proteins: Amide II/α-helical structure
		1488	Lipids: C-H deformation
1411	1407	1407	Lipids: C-H deformation
1373		1375	Lipids, proteins; C-N stretching of cytosin
		1280	Proteins: Amide III; collagen
1232	1238	1232	Phosphates: ν_asym_ PO^2-^
		1126	Carbohydrates: C-O stretching, RNA
1091	1085	1091	Phosphates: ν_sym_ PO^2-^
	1060		Carbohydrates: C-O stretching; DNA/RNA

Striking differences between the control groups and the sodium hypochlorite-treated MRSA were achieved with a very good degree of classification (Fig 5D and 5G). Despite being extensively studied, the mechanism of action of chlorine-based agents against bacteria has not been fully understood. It is hypothesized that a combination of factors including DNA breakage, inhibition of protein synthesis, membrane-associated damage, and oxidation of respiratory components can be conceived as a mode of action of sodium hypochlorite on bacteria [51–53]. Our results implicate the significant changes in the stretching modes of phosphates, deformation of lipids, and C = O ester in phospholipids, as sodium hypochlorite-induced damage to MRSA (Fig 5G). This finding clearly suggests damage to the phospholipid bilayers in the membrane. We achieved additional cellular alterations caused by sodium hypochlorite treatment including the amide I bands of proteins resulting from aggregated strands and turns, and the amide II band from α-helical structure of proteins (Table 3). This finding may suggest that MRSA membrane disruption and protein degradation are the sensitive targets affected by sodium hypochlorite.

Fig 5E shows the classification between triclosan-treated group and the control groups and the vector plot for this separation (Fig 5H) highlights the important discriminatory features. A very good degree of clustering between spectra from the triclosan-treated MRSA and the control groups was achieved (Fig 5H). Discriminatory features responsible for this classification are similar to the features in the comparison between alcohol-treated spectra and the control groups, albeit with slight differences (Table 3). The greatest difference was associated with the asymmetric stretching of phosphate (ν_asym_ PO_2_^−^) band. This band centered at 1238 cm^-1^ in the cluster vector plot associated with the triclosan-treated MRSA, but shifted to 1228 cm^-1^ in the vector plot of alcohol-treated MRSA. ν_asym_ PO_2_^−^ band is the main FTIR signature of the DNA conformational change by shifting from 1228 cm^−1^ in the B-DNA spectrum to 1238 cm^−1^ in the A-DNA spectrum [54–56]. This important shift suggests distinctive biochemical alterations in DNA conformations of MRSA induced by triclosan; a finding that clearly shows unique characteristics of triclosan and alcohols (ethanol and isopropanol) in inactivating MRSA. Results from this study, for the first time, demonstrate that although the alcohol- and the triclosan-induced damages to MRSA look alike; they may follow different regimens to modify DNA or RNA. However, more studies are needed for a better understanding of the overall mechanism of triclosan against MRSA.

Fig 5F shows a clustering of the triclocarban-treated spectra and the control groups. The triclocarban-treated spectra are dominated by absorptions from pure disinfectant. As demonstrated (Fig 5I), the most important differentiating bands lie in the protein region, lipid region, and nucleic acids region of the spectrum. Triclocarban-treated spectrum shows the most complex biochemistry among the disinfectants. The scores plot from comparing five treatments (Fig 5A) separates out the triclocarban-treated spectra from the other treatment. A comparison of the triclocarban-treated and the controls reveals alterations in the amide I and amide II band components resulting from aggregated strands, β-sheets, turns, and α-helical structures of proteins, deformation of lipids, phosphates, and C-O stretches attributable to carbohydrates and collagen (Fig 5I, Table 3). It has been hypothesized that the mode of action of triclocarban is similar to that of triclosan, and this involves cytoplasmic membrane disruption [47]. Although the major triclocarban- and triclosan-induced damage to MRSA lies in the protein region of the spectrum, there are major differences in the specific vibrational modes of the functional groups that are triggered by each disinfectant (Table 3). Though not completely clear, this finding is potentially important and may be beneficial in understanding the molecular and biological activity of MRSA in response to triclocarban.

## Conclusion

To date, this is the first time that FTIR spectroscopy combined with multivariate data analysis has been used to differentiate between untreated and the disinfectant-treated MRSA to reveal the underlying biomolecular alterations associated with each treatment. IR bands, arising from the symmetric and asymmetric stretching of phosphates that were sensitive against structural changes in phospholipids and DNA, was a common induced damage in all treatments. Moreover, the IR bands attributed to proteins and lipids were the major contributors to the damage induced by all biocides studied, and this is consistent with the fact that biocides react mostly with cytoplasmic constituents rather than nucleic acids. Our findings indicate that biospectroscopy can be used as a method to analyze bacterial cells at molecular levels and to yield objective results that show the mode of action and the bactericidal effects of disinfectants, particularly when other biochemical and pathological methods are limited. Further studies are needed to determine the precise action of biocides at molecular and cellular level. In addition, studies focused on the dose-dependent effects of biocides against MRSA will be of critical importance in enhancing our understanding of the interactions between hygiene products and bacterial cells.
